# Supplementary material for: Scalable interpolation of satellite altimetry data with probabilistic machine learning
Source: Nat Commun. 2024 Aug 28;15:7453. doi: 10.1038/s41467-024-51900-x (PMC11358133; doi:10.1038/s41467-024-51900-x)
Supplement: Supplementary file 1 — Supplementary Information [file 41467_2024_51900_MOESM1_ESM.pdf]

Scalable interpolation of satellite altimetry data with probabilistic machine learning

William Gregory*[1], Ronald MacEachern[2,3], So Takao[2], Isobel R. Lawrence[4], Carmen Nab[3,5], Marc Peter Deisenroth[2,6], and Michel Tsamados[3]

[1]Atmospheric and Oceanic Sciences Program, Princeton University, Princeton, USA

[2]UCL Centre for Artificial Intelligence, University College London, London, UK

[3]Centre for Polar Observation and Modelling, University College London, London, UK

[4]ESRIN, European Space Agency, Frascati, Italy

[5]Ocean Forecasting Research & Development, Met Office, Exeter, UK

[6]The Alan Turing Institute, London, UK

* Correspondence: William Gregory (wg4031@princeton.edu)

1 Methods: GPSat details

In this supplementary information, we provide further details on the various components of GPSat.

1.1 Background on Gaussian processes

A Gaussian process (GP) is a random function $f : \mathbb{R}^n \to \mathbb{R}$ such that for any set of points $\boldsymbol{x}_1, \ldots, \boldsymbol{x}_N \in \mathbb{R}^n$, the random variable $\boldsymbol{f} := (f(\boldsymbol{x}_1), \ldots, f(\boldsymbol{x}_N))^\top \in \mathbb{R}^N$ is multivariate Gaussian, i.e., $\boldsymbol{f} \sim \mathcal{N}(\boldsymbol{\mu}, \boldsymbol{\Sigma})$ for some $\boldsymbol{\mu} \in \mathbb{R}^N$ and $\boldsymbol{\Sigma} \in \mathbb{R}^{N \times N}$. Equivalently, we can characterise GPs by a *mean function* $m : \mathbb{R}^n \to \mathbb{R}$ and a *kernel* $k : \mathbb{R}^n \times \mathbb{R}^n \to \mathbb{R}$; the latter is a symmetric function with the property that for any set of points $\boldsymbol{x}_1, \ldots, \boldsymbol{x}_N \in \mathbb{R}^n$, the matrix $\mathbf{K}_{\mathbf{XX}} \in \mathbb{R}^{N \times N}$ whose (i, j)-th entry is given by $k(\boldsymbol{x}_i, \boldsymbol{x}_j)$ is positive definite. Indeed, we can relate the two definitions by setting $\boldsymbol{\mu} = (m(\boldsymbol{x}_1), \ldots, m(\boldsymbol{x}_N))^\top$ and $\boldsymbol{\Sigma} = \mathbf{K}_{\mathbf{XX}}$.

From a practical point of view, GPs are useful since they can be used to interpolate labelled data $\{(\boldsymbol{x}_n, y_n)\}_{n=1}^N$ at new inputs $\{\boldsymbol{x}_n^*\}_{n=1}^{N^*}$ through Bayes' rule, providing a principled and robust way to assimilate data. This has the additional advantage that the predictions come equipped with uncertainties, which we do not necessarily get from other interpolation methods. Concretely, given a dataset $\{(\boldsymbol{x}_n, y_n)\}_{n=1}^N$, the predictions $\boldsymbol{f}^* = (f(\boldsymbol{x}_1^*), \ldots, f(\boldsymbol{x}_{N_*}^*)) \in \mathbb{R}^{N_*}$ at new input locations $\{\boldsymbol{x}_n^*\}_{n=1}^{N_*}$ is a Gaussian $\boldsymbol{f}^*|\boldsymbol{y} \sim \mathcal{N}(\boldsymbol{\mu}^*, \boldsymbol{\Sigma}^*)$, whose mean and covariance are computed as

$$\boldsymbol{\mu}^* = m(\mathbf{X}^*) + \mathbf{K}_{*\mathbf{X}}(\mathbf{K}_{\mathbf{XX}} + \sigma^2\mathbf{I})^{-1}(\boldsymbol{y} - m(\mathbf{X})) \in \mathbb{R}^{N_*}, \tag{1}$$

$$\boldsymbol{\Sigma}^* = \mathbf{K}_{**} - \mathbf{K}_{*\mathbf{X}}(\mathbf{K}_{\mathbf{XX}} + \sigma^2\mathbf{I})^{-1}\mathbf{K}_{\mathbf{X}*} \in \mathbb{R}^{N_* \times N_*}, \tag{2}$$

where we denoted by $\mathbf{K}_{\mathbf{XX}} \in \mathbb{R}^{N \times N}, \mathbf{K}_{*\mathbf{X}} \in \mathbb{R}^{N_* \times N}$ and $\mathbf{K}_{**} \in \mathbb{R}^{N_* \times N_*}$, the matrices whose (i, j)-th entry is given by $k(\boldsymbol{x}_i, \boldsymbol{x}_j), k(\boldsymbol{x}_i^*, \boldsymbol{x}_j)$ and $k(\boldsymbol{x}_i^*, \boldsymbol{x}_j^*)$, respectively. To understand GP uncertainties here, it is worth revisiting Bayes' rule. This states that the predicted posterior Gaussian distribution is proportional to the product of two independent Gaussian distributions, $p(\boldsymbol{f}^*|\boldsymbol{y}) \propto p(\boldsymbol{y}|\boldsymbol{f}^*)p(\boldsymbol{f}^*)$. In this context, $\boldsymbol{y}|\boldsymbol{f}^* \sim \mathcal{N}(\boldsymbol{f}^*, \sigma^2\mathbf{I})$ is the *likelihood* distribution describing the probability of the observations given our GP model, and $\boldsymbol{f}^* \sim \mathcal{N}(\boldsymbol{\mu}, \boldsymbol{\Sigma})$ describes our *prior* belief about the data-generating function, independent of any observations. The relative weight assigned to the prior and likelihood distributions in Bayes' rule largely depends on the number of observations present. In the case where there are no observations, the posterior distribution is equal to the prior, having mean and covariance $m(\mathbf{X}^*)$ and $\mathbf{K}_{**}$, respectively. Conversely, as the observations fill up the entire domain, the posterior will concentrate around the ground truth with vanishing covariance. This is known as posterior consistency [1].

The choice of kernel is important in defining a GP (usually more so than the mean function), as it determines the properties of the function f being modelled. As a standard example, the squared-exponential kernel is given by

$$k(\boldsymbol{x}, \boldsymbol{x}') = \tau^2 \exp\left(-\frac{\|\boldsymbol{x} - \boldsymbol{x}'\|^2}{2\ell^2}\right), \tag{3}$$

which produce predictions that are very smooth (technically, infinitely differentiable). The parameters τ and ℓ, referred to as the amplitude and lengthscale hyperparameters, control the variability and spatial correlation of the process, respectively. A more popular choice in spatial statistics is the Matérn kernel, given by

$$k(\boldsymbol{x}, \boldsymbol{x}') = \tau^2 \frac{2^{1-\nu}}{\Gamma(\nu)} \left(\sqrt{2\nu}\frac{\|\boldsymbol{x} - \boldsymbol{x}'\|}{\ell}\right)^\nu K_\nu \left(\sqrt{2\nu}\frac{\|\boldsymbol{x} - \boldsymbol{x}'\|}{\ell}\right), \tag{4}$$

where K_ν for $\nu > 0$ is the modified Bessel function of the second kind, Γ is the Gamma function, and σ, ℓ are again the amplitude and lengthscale hyperparameters of the process respectively. The parameter ν in the Matérn kernel allows control over the smoothness of the predictions, with smaller ν leading to rougher predictions and larger ν leading to smoother predictions (in fact, it recovers the squared-exponential kernel in the limit $\nu \to \infty$). This added flexibility on the smoothness makes it ideal in various settings, as spatial fields in the real world typically exhibit some degree of roughness.

The Bayesian nature of GPs allows one to identify in a principled way, which kernel is the most suitable for interpolating a given dataset. In particular, we can infer the choice of hyperparameters for a given kernel type that are most suitable for modelling the data, which we discuss in the next section.

1.2 Training step

In GP modelling, we refer to "training" as the procedure of learning the hyperparameters of the GP model that best fit the training data without overfitting. In GPSat, we let each local GP expert κ carry their own set of hyperparameters $\boldsymbol{\theta}_\kappa$ and use Type-II maximum likelihood estimation[2] to get estimates of the optimal $\boldsymbol{\theta}_\kappa$. This proceeds by minimising the loss

$$\mathcal{L}[\boldsymbol{\theta}_\kappa] = \frac{1}{2}(\boldsymbol{y} - m(\mathbf{X}))^\top [\mathbf{K}_{\mathbf{XX}}(\boldsymbol{\theta}_\kappa) + \sigma_\kappa^2 \mathbf{I}]^{-1}(\boldsymbol{y} - m(\mathbf{X})) + \frac{1}{2}\log\left|\mathbf{K}_{\mathbf{XX}}(\boldsymbol{\theta}_\kappa) + \sigma_\kappa^2 \mathbf{I}\right|, \quad (5)$$

where $\boldsymbol{y} = (y_1, \ldots, y_{N_\kappa})^\top$ are the observations inside the training region and as before, $\mathbf{K}_{\mathbf{XX}}(\boldsymbol{\theta}_\kappa) = k(\mathbf{X}, \mathbf{X}; \boldsymbol{\theta}_\kappa)$ denotes the covariance of the local GP at locations $\mathbf{X} = (\boldsymbol{x}_1, \ldots, \boldsymbol{x}_{N_\kappa})^\top$ where the observations are made. The parameter σ_κ is the standard deviation of the Gaussian observation error, which may be learned as well. Minimising (5) can be achieved through standard gradient-based methods, such as L-BFGS. Moreover, using modern machine learning frameworks, such as Tensorflow and PyTorch, the gradients can be computed via automatic differentiation, and the linear algebra computations can be accelerated by utilising GPUs.

As a technical sidenote, in order to prevent the hyperparameters from taking unreasonably small or large values, we apply constraints during training so that its values do not exceed specified lower and upper bounds, say θ_{min} and θ_{max}. The constraints are applied by transforming a set of "raw" hyperparameters $\theta^i_{\text{raw}} \in \mathbb{R}$ by a scaled and shifted sigmoid function, so that $\theta^i := \text{Sigmoid}(\theta^i_{\text{raw}}) \in [\theta_{\text{min}}, \theta_{\text{max}}]$. Note that we train the raw hyperparameters θ^i_{raw} instead of the actual model hyperparameters θ^i here.

1.3 Inference step

Once the model is trained and we have the hyperparameters $\boldsymbol{\theta}_\kappa$ that are optimal in the sense of Type-II maximum likelihood estimation, we can infer the posterior distributions $p(f_\kappa(\boldsymbol{x}^*)|\boldsymbol{y}_\kappa)$ of each local expert GP f_κ evaluated at arbitrary test locations $(\boldsymbol{x}^*_1, \ldots, \boldsymbol{x}^*_{N_*})$ within the inference region of expert κ (here, $\boldsymbol{y}_\kappa$ denotes all the data points inside the training region of the expert κ). This is computed using (1)–(2) for each κ.

1.4 Post-processing

Below, we describe two post-processing procedures that we employ in GPSat to generate our final predictions, namely, gluing of local predictions and hyperparameter smoothing.

1.4.1 Gluing local predictions

After performing inference at all expert locations, we require a procedure to combine all of the local predictions into a single global prediction. For this purpose, we first introduce a partition-of-unity $\{w_\kappa(\boldsymbol{x})\}_{\kappa=1}^K$ (i.e., a family of smooth functions over $\mathbb{R}^n$ that have compact support and which satisfies the condition $\sum_{\kappa=1}^K w_\kappa(\boldsymbol{x}) = 1$ for every $\boldsymbol{x} \in \mathbb{R}^n$). Then, for

any $\boldsymbol{x}^* \in \mathbb{R}^n$, we glue our local predictions $\{p(f_\kappa(\boldsymbol{x}^*)|\boldsymbol{y}_\kappa)\}_{\kappa=1}^K$ together by taking a linear combination as follows:

$$p(f(\boldsymbol{x}^*)|\boldsymbol{y}) \approx \sum_{\kappa=1}^K w_\kappa(\boldsymbol{x}^*)\, p(f_\kappa(\boldsymbol{x}^*)|\boldsymbol{y}_\kappa). \tag{6}$$

In practice, we employ moment-matching to obtain a Gaussian approximation to the RHS of (6). For example, if the local predictions $p(f_\kappa(\boldsymbol{x}^*)|\boldsymbol{y}_\kappa)$ are Gaussians with central moments $(\mu_\kappa^*, (\sigma_\kappa^*)^2)$, then we approximate (6) by a Gaussian $\mathcal{N}(\mu^*, (\sigma^*)^2)$ with

$$\mu^* := \sum_{\kappa=1}^K w_\kappa(\boldsymbol{x}^*)\mu_\kappa^*, \qquad (\sigma^*)^2 := \sum_{\kappa=1}^K w_\kappa(\boldsymbol{x}^*)(\sigma_\kappa^*)^2. \tag{7}$$

That is, we simply compute a weighted average of the means and variances with respect to the weights $\{w_\kappa(\boldsymbol{x}^*)\}_{\kappa=1}^K$.

For the choice of our partition-of-unity $\{w_\kappa(\boldsymbol{x})\}_{\kappa=1}^K$, we consider the functions

$$w_\kappa(\boldsymbol{x}) := \frac{\mathcal{N}_r(\boldsymbol{x}|\boldsymbol{x}_\kappa, \sigma)}{\sum_{\kappa=1}^K \mathcal{N}_r(\boldsymbol{x}|\boldsymbol{x}_\kappa, \sigma)}, \qquad \kappa = 1, \ldots, K, \tag{8}$$

where $\mathcal{N}_r(\boldsymbol{x}|\boldsymbol{x}_\kappa, \sigma)$ is a truncated Gaussian density with support on $B_r(\boldsymbol{x}_\kappa) := \{\boldsymbol{x} \in \mathbb{R}^n : \|\boldsymbol{x} - \boldsymbol{x}_\kappa\| < r\}$, where $r > 0$ is the radius of the inference region and $\boldsymbol{x}_\kappa$ is the local expert location (i.e., the central point of the training and inference regions). Note that strictly, this does not satisfy our assumption that w_κ is a smooth function in $\mathbb{R}^n$ due to the discontinuity at the boundary $\partial B_r(\boldsymbol{x}_\kappa) := \{\boldsymbol{x} \in \mathbb{R}^n : \|\boldsymbol{x} - \boldsymbol{x}_\kappa\| = r\}$. However, we can make this discontinuity negligibly small by choosing a small enough $\sigma > 0$. For example, choosing $\sigma = r/3$, we can ensure that $\approx 99\%$ of the total mass of $w_\kappa(\boldsymbol{x})$ is contained inside $B_r(\boldsymbol{x}_\kappa)$, making this discontinuity insignificant.

1.4.2 Hyperparameter smoothing

We sometimes encounter large differences in the learned hyperparameters between nearby expert locations, which can lead to poor reconstructions of the global field upon gluing the local predictions. In addition, optimisation may fail occasionally, producing `NaN` values for the learned hyperparameters at some expert locations. In order to overcome these issues, after the training step, we may opt to perform smoothing of the hyperparameters $\{\boldsymbol{\theta}_\kappa\}_{\kappa=1}^K$ before proceeding to the inference step. We achieve this by a simple kernel smoothing

$$\boldsymbol{\theta}_\kappa^{\text{new}} = \frac{\sum_{m=1}^K k(\boldsymbol{x}_m, \boldsymbol{x}_\kappa)\boldsymbol{\theta}_m}{\sum_{m=1}^K k(\boldsymbol{x}_m, \boldsymbol{x}_\kappa)}, \quad \text{for all} \quad \kappa = 1, \ldots, K, \tag{9}$$

for a choice of smoothing kernel k, which we choose to be squared-exponential, i.e.,

$$k(\boldsymbol{x}, \boldsymbol{x'}) = \exp(-\|\boldsymbol{x} - \boldsymbol{x}'\|^2/2s^2). \tag{10}$$

Here, $s > 0$ is a parameter to be tuned, controlling the smoothness of the hyperparameter field. In the computation (9), we omit any parameters $\boldsymbol{\theta}_\kappa$ whose values are `NaN`s. These get updated to new values $\boldsymbol{\theta}_\kappa^{\text{new}}$, which are not `NaN`s through kernel smoothing.

1.5 Sparse Gaussian processes

We have found that GPSat scales to spatial resolutions of around 5 km^2 and any resolutions beyond this result in significantly increased run time or exhaustion of GPU memory. In order to overcome this, we have considered the use of *sparse Gaussian processes*[3;4], which is an approximate inference method that enables one to effectively use GPs with over millions of data points. This proceeds by transferring the information contained in the original dataset to a set of M pseudo-data points, referred to as the *inducing variables*, and performing training and inference with respect to these pseudo-data instead of the original data. By choosing $M \ll N$ inducing variables, where N is the size of the original dataset, we can reduce the computational costs substantially, from $\mathcal{O}(N^3)$ to $\mathcal{O}(NM^2 + M^3)$, and memory cost from $\mathcal{O}(N^2)$ to $\mathcal{O}(NM + M^2)$.

Intuitively, this method is effective when the original dataset contains a high level of redundancy; for example when the data is sampled at very high frequency, but the information received from consecutive data points are not significantly different. In this case, one would expect that the GP would perform just as well if tight clusters of data points were to be each represented by single data points. Sparse GPs therefore provide a principled way to effectively reduce the number of data points without actually discarding them from the dataset, as done using e.g., Nyström approximation[2]. The latter may lead to poor results as we have no guarantee that the sub-sampled data is necessarily representative of the original dataset.

Concretely, the learning of the M inducing variables is achieved by variational inference, which computes an approximate posterior $q(\boldsymbol{f}, \boldsymbol{f}^*) \approx p(\boldsymbol{f}, \boldsymbol{f}^*|\boldsymbol{y})$ by minimising the Kullback-Leibler (KL) divergence $\mathcal{KL}(q(\cdot)||p(\cdot|\boldsymbol{y}))$ over q in some variational family, such as the family of Gaussians. In sparse GPs, the variational distribution $q(\boldsymbol{f}, \boldsymbol{f}^*)$ is constructed from $M \ll N$ inducing variables $\boldsymbol{u} = f(\boldsymbol{z})$ by the relation

$$q(\boldsymbol{f}, \boldsymbol{f}^*) = \int p(\boldsymbol{f}|\boldsymbol{u})p(\boldsymbol{f}^*|\boldsymbol{u})q(\boldsymbol{u})\mathrm{d}\boldsymbol{u}, \tag{11}$$

assuming conditional independence between the variables $\boldsymbol{f}$ and $\boldsymbol{f}^*$ given $\boldsymbol{u}$. Here, $q(\boldsymbol{u})$ is a multivariate Gaussian on $\mathbb{R}^M$, whose mean vector $\boldsymbol{m} \in \mathbb{R}^M$ and covariance matrix $\mathbf{S} \in \mathbb{R}^{M\times M}$ parameterise the variational distribution. These are in turn determined by minimising the KL divergence objective, or equivalently, to maximise the evidence lower bound (ELBO), which is more tractable computationally. When the likelihood $p(\boldsymbol{y}|\boldsymbol{f})$ is Gaussian, then we can compute a closed form expression for the parameters $\boldsymbol{m}$ and $\mathbf{S}$ that minimise the KL divergence[3]. This leads to the Sparse Gaussian process regression (SGPR) algorithm, which

has the computational complexity of $\mathcal{O}(NM^2 + M^3)$ and memory cost of $\mathcal{O}(NM + M^2)$, enabling one to use moderately large datasets of up to $\mathcal{O}(10^5)$ data points, by choosing M to be small enough.

On the other hand, when the likelihood $p(\boldsymbol{y}|\boldsymbol{f})$ is non-Gaussian, or the number of data points are in the millions, we can use the Sparse variational Gaussian process (SVGP) algorithm [4], which learn the parameters $\boldsymbol{m}$ and $\mathbf{S}$ by maximising the ELBO using stochastic gradient-based optimisation. By using a mini-batch size of $B \ll N$, we can reduce the computational complexity to $\mathcal{O}(BM^2 + M^3)$ and the memory cost to $\mathcal{O}(BM + M^2)$, enabling one to use over millions of data points. There are trade-offs however, and careful tuning of the mini-batch size and the number of inducing variables is necessary – using more inducing variables will generally lead to more accurate results, however this must be compensated by using smaller mini-batches, which leads to noisier and slower training process.

There are other acceleration methods for GP training and inference such as Scalable Kernel Interpolation [5], inter-domain methods [6;7;4], SPDE-INLA [8] and use of randomised numerical linear algebra methods [9], however we leave the investigation and comparions of these approaches for future work.

2 Results: GPSat sensitivity analysis

**Training Window Configuration**	**RMSD (cm)**	**R^2**	**Runtime (hours)**
± 300 km / ± 4 days	10.2	0.39	11.6
± 300 km / ± 3 days	10.2	0.39	10.4
± 300 km / ± 2 days	10.3	0.38	6.0
± 300 km / ± 1 day	10.3	0.37	5.2
± 300 km / ± 0 days (2-D)	10.5	0.35	1.2
± 200 km / ± 4 days	10.2	0.39	6.1
± 200 km / ± 3 days	10.2	0.39	5.3
± 200 km / ± 2 days	10.2	0.38	5.1
± 200 km / ± 1 day	10.3	0.37	4.6
± 200 km / ± 0 days (2-D)	10.6	0.34	1.1
± 100 km / ± 4 days	10.2	0.38	4.4
± 100 km / ± 3 days	10.3	0.38	4.2
± 100 km / ± 2 days	10.3	0.37	4.1
± 100 km / ± 1 day	10.5	0.36	4.0
± 100 km / ± 0 days (2-D)	11.0	0.29	0.8

Table 1: Repeated cross-validation analysis of 5 km-gridded GPSat radar freeboard predictions. Here Sentinel-3A (S3A) tracks are withheld on each day over December 2018, and used for validation. Prediction metrics and runtimes are reported for different training window sizes. See section 2.3 and Fig. 6 of the main article for further details of cross-validation approach.

References

[1] Choi, T. & Schervish, M.J. On posterior consistency in nonparametric regression problems. *Journal of Multivariate Analysis* **98,** 1969–1987 (2007).

[2] Williams, C. & Rasmussen, C.E. Gaussian processes for machine learning. *MIT press Cambridge* (2006).

[3] Titsias, M. Variational learning of inducing variables in sparse Gaussian processes. *Proceedings of Machine Learning Research* **5,** 567–574 (2009).

[4] Hensman, J., Fusi, N. & Lawrence, N.D. Gaussian processes for big data. Preprint at https://arxiv.org/abs/1309.6835 (2013).

[5] Wilson, A. & Nickisch, H. Kernel interpolation for scalable structured Gaussian processes (KISS-GP). *Proceedings of Machine Learning Research* **37,** 1775–1784 (2015).

[6] Cunningham, H.J., et al. Actually Sparse Variational Gaussian Processes. *International Conference on Artificial Intelligence and Statistics* 10395–10408 (2023).

[7] Dutordoir, V., Durrande, N. & Hensman, J. Sparse Gaussian processes with spherical harmonic features. *International Conference on Artificial Intelligence and Statistics* 2793–2802 (2020).

[8] Lindgren, F. & Håvard, R. Bayesian Spatial Modelling with R-INLA. *Journal of statistical software* **63,** (2015).

[9] Mahoney, M.W. Randomized algorithms for matrices and data. *Foundations and Trends in Machine Learning* **3,** 123–224 (2011).
